# Developing a Weight-Neutral Health Intervention in Denmark: Protocol for a Co-Design Process

**DOI:** 10.2196/59455

**Published:** 2024-11-29

**Authors:** Lene Meyer, Rasmus Køster-Rasmussen, Ann-Kathrin Lindahl Christiansen, Berit Lilienthal Heitmann, Gitte Kingo Vesterlund, Sofus Christian Larsen, Ina Olmer Specht, Fanney Thorsteinsdottir, Emma Katrine Frøhlke Steinbo, Clara Lundmark Appel, Gudrun Agusta Sigurdardottir, Susanne Reventlow, Frans Boch Waldorff, Catharina Thiel Sandholdt

**Affiliations:** 1 Research Unit for General Practice and Section of General Practice, Department of Public Health, University of Copenhagen, Øster Farimagsgade 5, Copenhagen, 1353, Denmark, 45 31646460; 2 Research Unit for Dietary Studies at The Parker Institute, Frederiksberg and Bispebjerg Hospital, Frederiksberg, Denmark

**Keywords:** obesity, weight management, complex intervention, patient engagement, health intervention, Denmark, Danish, lifestyle, weight loss, weight reduction, stigma, neutral health, human-centered design, dissemination, podcast, social media, feasibility study, public health, surveillance

## Abstract

**Background:**

Lifestyle interventions for weight loss are generally ineffective in achieving clinically meaningful long-term reductions in body weight and may contribute to negative behavior such as weight cycling or disordered eating. Negative focus on high weight may also contribute to weight stigma. Weight stigma includes negative attitudes and discriminatory behavior toward people with big bodies and can result in psychological stress and unfavorable health outcomes. Taken together, it is possible that the potential harms of lifestyle-based weight loss interventions may exceed the potential benefits. Weight-neutral health (WNH) has emerged as an alternative strategy advocating for size diversity, intuitive eating, and joyful physical movement, all without placing emphasis on weight reduction.

**Objective:**

This protocol outlines the study design for the co-design process of developing a WNH complex intervention, engaging relevant stakeholders in Denmark.

**Methods:**

We base our understanding of WNH on the principles from Health at Every Size: body acceptance, joyful movement, intuitive eating, and weight stigma reduction. The co-design development process is based on the Medical Research Council’s framework for complex interventions and applies methods from human-centered design through 4 iterative design phases of engaging stakeholders—*discover*: search existing literature, and conduct interviews with Danish municipal stakeholders working with WNH and other expert stakeholders; *define*: coproduction of seminars with health professionals (HPs) with knowledge of WNH, and semistructured interviews with people with BMI≥30 kg/m^2^ who have participated in existing WNH interventions; *design*: content-creating workshops with HPs and people with BMI≥30 kg/m^2^; and *validat*e: evaluate seminars, plan feasibility, and produce materials. The data will be analyzed thematically to build a scaffold for the intervention activities and components. In further analysis, we will explore how health is performed, meaning the actions and dialogues that arise when dealing with health guidelines, the societal body, weight, and health expectations, in the context of the intervention.

**Results:**

The project is fully funded. As of August 2024, the co-design process was in the closing phase. In total, 15 HPs were included, some of whom have larger body sizes. This provides a dual perspective, combining their personal experiences of living with a high BMI with their professional expertise. In total, 16 people with BMI≥30 kg/m^2^ have generously shared their experiences with WNH programs, including the difficulties of moving away from external demands and personal wishes for weight loss. Their contributions have nuanced and unfolded our understanding of the principles of WNH in a Danish setting.

**Conclusions:**

The intervention designed in and from the co-design process will be tested for feasibility in 2025. The findings from the feasibility study will inform a future randomized controlled trial and present novel findings in the field of health management. The long-term goal is to implement the intervention in a Danish municipal setting free of charge.

## Introduction

The rising number of people with a BMI above 30 kg/m^2^ is considered a public health concern. In Denmark, health authorities promote weight loss through lifestyle changes for individuals with a BMI>30 kg/m^2^ and encourage municipal health units to offer weight loss programs to these citizens [1]. Weight loss recommendations in the US begin at a BMI of 25 kg/m^2^ depending on the presence of co-morbidities [2,3]. However, systematic evaluations find that lifestyle interventions for weight loss are generally ineffective in achieving clinically meaningful long-term reductions in body weight [4-6]. Other studies suggest that weight loss interventions may come with adverse consequences for individuals since dieting can lead to weight cycling, self-criticism, and disordered eating [7-10]. Furthermore, weight loss interventions seldom, if ever, address potential harms such as weight stigma and may themselves contribute to weight stigma by focusing on high body weight as something wrong. Weight stigma is widespread in health care and society [11]. Weight bias and weight stigma include negative attitudes toward and stereotypes about people in larger bodies, which can lead to discriminatory behaviors [12]. Weight stigma may result in psychological stress and has been linked to a variety of unfavorable health outcomes [13-15]. Most researchers in the field of body weight, international obesity societies, and patient organizations agree that weight stigma is a health issue that must be addressed [16]. Some prospective studies link weight stigma to long-term declines in self-reported health, increased maladaptive eating behaviors, weight gain [17], and potentially death [18]. Weight stigma is a predictor of poor mental and physical health, and these associations are generally independent of sociodemographic variables and baseline BMI [19-24].

Taken together, it is possible that the potential harms of lifestyle-based weight loss interventions may exceed the potential benefits, especially given the poor prognosis of long-term weight loss management. Furthermore, weight stigma may account for a substantial amount of the unfavorable health outcomes associated with high BMI [18-24]. As a result, it is worthwhile investigating approaches to improve health in individuals with a high BMI that do not focus on weight loss but do address weight stigma.

In recent years, weight-neutral health (WNH) has gained prominence as a holistic approach to health, advocating for size diversity, intuitive eating, and enjoyable physical movement, all without placing emphasis on weight reduction [25]. There are, however, few and only small studies that have tested the effects of WNH interventions with a short follow-up period [26,27]. Earlier WNH interventions have focused on decreasing weight self-stigma, emotional eating, shame, and self-criticism [26,27]. The interventions differ, but most studies have used acceptance-, mindfulness-, and compassion-based group interventions and most of them have demonstrated reductions in internalized weight bias and improvements in mental health and quality of life [28-30]. A meta-analysis of 9 smaller controlled studies of WNH interventions from North America compared WNH interventions to weight-loss or weight-control interventions and found no differences in body weight and cardiovascular risk factors after 3-12 months of follow-up between the 2 strategies, but fewer participants developed bulimic symptoms in the WNH interventions [26]. Thus, the scientific literature indicates that weight neutrality can be a viable alternative to the weight-loss paradigm, but larger studies are needed, including further research that explores a real-life setting as well as participant motivations for participating in weight-neutral interventions [31]. This protocol will present the co-design process for developing a weight-neutral intervention in a Danish context (WIN), which aims to create a WNH intervention for people with a high BMI (BXs) that (1) is implementable in a primary health care context; (2) may reduce internalized weight stigma and improve mental, social, and physical health; and (3) is perceived as meaningful to the target groups. The WIN intervention will go on to be tested in a feasibility study where it will be assessed on recruitment, treatment fidelity, and adherence in a non-controlled setting.

## Methods

This protocol outlines the co-design process of the WIN intervention.

The WIN development process will be founded on weight-neutral principles of Health At Every Size [25], which include (1) body acceptance, (2) intuitive eating, (3) joyful movement, and (4) weight stigma reduction. Weight reduction is not an aim. The WIN co-design development process will be based on active participation and the involvement of end users, ie, those who deliver and receive the intervention. Therefore, relevant health professionals (HPs), BXs, municipal stakeholders, and weight researchers will be included in the process.

The development process is aligned with the MRC framework for complex interventions [32] and we apply principles of the human-centered design tradition to organize the development process into 4 temporal iterative phases—*discover*, *define*, *design*, and *validate*—each with a specific focus of understanding and answering the issue of concern [33-36]. Additionally, the process aligns with the British INVOLVE framework, which states that “no decision about me [should be made] without me” [37].

A primary study line will explore the experiences and challenges of HPs who provide weight-neutral therapies. A secondary path will explore the experiences and challenges of individuals with a high BMI (BX participants) who have taken part in a WNH intervention. Both paths will follow a practice theory approach [38-40]. We will examine these practices in relation to existing norms, internalized weight stigma, and the participants’ (HPs and BXs) conception of what constitutes a healthy body and lifestyle [41,42].

The core WIN research team includes 3 general practitioners (RKR, GAS, and FBW), a psychologist (LM), and a health sociologist (CTS). Two are both researchers and practicing clinicians (LM and RKR). In total, 3 members have substantial knowledge about the WNH paradigm (LM, RKR, and GAS). In co-designing the WIN intervention, we as researchers participate in the process of producing insights and solutions, and we are aware of our roles, assumptions, and body privileges [43,44]. In the core WIN research team, we all have body sizes that allow us to move freely in society without having to fear weight-based judgements or discrimination. We acknowledge that weight stigma and thin privilege are two sides of the same coin and that weight stigma experiences of people in larger bodies will be largely invisible to us [45,46]. We will constantly seek to challenge our privileges and acknowledge and respect the experiences that are shared with us. This process extends beyond the project and into our own lives. The core team receives active support from a larger group of interdisciplinary researchers in the LightCOM (Lighthouse Consortium on Obesity Management) project.

### Study Setting

The WIN intervention will be developed and implemented in Denmark and adapted to the legislative role that municipalities have in primary healthcare health promotion. Danish municipalities already provide a variety of health promotion initiatives, including weight-reduction courses and a few weight-neutral aligned courses [47]. The WIN intervention will be designed to be integrated into existing municipal health units across the country with built-in flexibility to accommodate the unique conditions of each municipality in terms of demography and accessibility.

### Study Population

In this co-design study, the study population consists of selected end-user representatives:

1. Stakeholders and HPs from 3‐7 health departments in the municipalities with weight-neutral aligned courses. Denmark consists of 98 municipalities, with few offering weight-neutral programs. We therefore wish to map the municipalities working with the Health at Every Size (HAES) principles and how this is implemented to learn about the reflections, dilemmas, and experiences that they have given rise to.
2. In total, 10‐15 HPs from a range of public and private health care settings who have experience with WNH practices. The HPs will be close collaborators in the development process, and the research team is dependent on their experiences and expertise. We aim to include 10‐15 HPs to form a group of diversity in training and geography. This group size is suitable for working in smaller groups as well as engaging in plenary discussions. The group is also sufficiently large to endure potential drop-outs and occasional absences.
3. In total, 5‐10 BXs (BMI ≥30 kg/m^2^) who have participated in existing WNH programs over the last 2 years. BXs are the key stakeholders who represent potential receivers of the intervention. We will include their experiences of participating in a weight-neutral program with a focus on their motivation and experiences of enablers and barriers during the program. Our analysis of their contribution is thus very focused, which is why we will only include a smaller number of participants compared with the HPs [48].

### Study Design and Patient and Public Involvement Strategy

**Figure 1.**
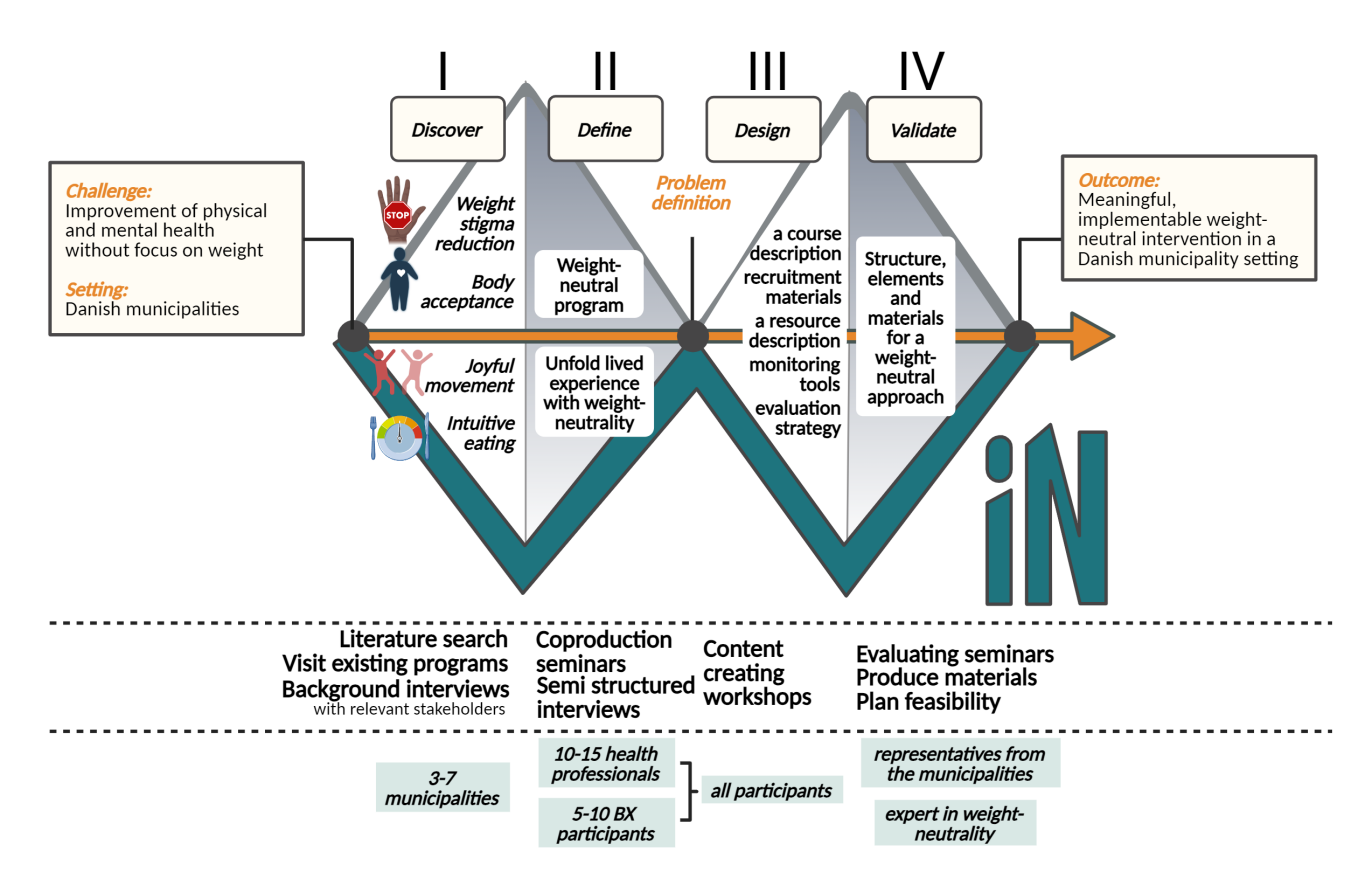
WIN study design: 4 phases presenting the codesign process in a double-diamond figure. The figure’s top bar depicts how we operate iteratively, beginning our research in phases I and II and tying knots and making project decisions in phases II and IV. The middle bar lists the methodologies used while the bottom bar includes the stakeholders involved in each phase. BX: people with a high BMI; WIN: weight-neutral intervention.

The co-design process is scheduled to last 15 months and will adhere to the human-centered design tradition framework with 4 iterative design phases: *discover, define, design,* and *validate* [33] (see Figure 1). The *discover* and *define* phases operate within a problem area and seek to define the specific issues that the intervention must solve. The *design* and *validate* phases take place in a solution area where the intervention’s specifics are designed. Figure 1 displays the 4 phases of the process. Between phases, the research group will have iteration meetings to document new insights and discuss the implications of these for the development and implementation of the WIN intervention. This will result in a formative evaluation procedure, qualifying the future feasibility study of the intervention. The various phases and iterative motions between being open and explorative, narrowing the focus, and making decisions are part of a developmental process in which we identify the problems and propose practical solutions. The 4 phases are described in detail below.

### Phase I: Discover

The first phase aims to explore and map the problems and opportunities summarized in the introduction section of this article. We will review the literature on WNH interventions, weight stigma, and body image. The evidence of interest includes biomedical and epidemiological science about weight and health as well as cultural and psychological studies on perceptions, experiences, and contextual factors. We will visit 3‐7 municipalities that have, are considering, or are currently conducting a WNH intervention. We will have meetings with relevant stakeholders, including weight-neutral therapists and staff managers, and perform interviews with people who have already participated in a WIN.

All participants will be recruited as a convenience sample using social media and the research team’s professional network.

### Phase II: Define

In the second phase, in collaboration with the target groups, we narrow our focus and define the core challenges that we want to address in the WIN intervention.

#### Activities With HPs

A total of 10‐15 HPs are invited to participate in a 16-week co-design program that includes full-day seminars every 3 weeks and 2-hour online meetings in between (a total of 87 h). The program will disseminate knowledge about WNH principles and methods, so the HPs have a shared knowledge foundation and have inspiration for the co-design process. The program will integrate the HPs’ skills, experiences, and reflections related to working with a weight-neutral approach, as well as their existing preconceptions. The purpose is to encourage a collaborative and nuanced study of how the intervention might be structured.

Throughout the program, the participating HPs will be grouped into pairs of 2 and asked to write letters or emails to each other between sessions to document their reflections and personal processes. They will be provided with a template to write from. Following each full-day seminar, participants will be asked to submit a brief questionnaire on their day’s output and engagement for formative assessment purposes.

#### Interviews With BXs

We will conduct semistructured interviews with 5‐10 participants from existing publicly supported municipal weight-neutral programs [49]. The inquiries will focus on the informant’s history, their first encounter with WNH, specifics about the program they have participated in, and the impact it had on their daily life and habits. The overall aim is to produce knowledge about enablers and barriers to participation, including motivational factors. The interview narratives and quotes will be used in the co-design sessions with HPs.

### Phase III: Design

The design phase will be centered around 2 content-creating workshop sessions. The first workshop will be distributed over 2 days with the HPs from phase II, where they will aim to develop an overarching goal and vision for the intervention as well as specifics for the various elements included in the intervention. This work will build directly on the work done in phase II’s co-design sessions.

In the second workshop, BXs will work with the preliminary findings from phase II and provide knowledge and experiences that will inform and shape the key principles and elements of the intervention. This will also serve to highlight situations or intervention themes, which can be particularly challenging or vulnerable to the target group.

Photos, citations, excerpts from researcher field notes, video clips, excerpts from assessment letters, and other materials may be utilized in the workshops. The materials will be decontextualized by the WIN researchers and will serve as building blocks in the workshop [48]. The goal is to design and establish the framework and essential parts of the WIN intervention, as well as to produce an inspiration catalog with optional methods and activities for HPs who will deliver it. The co-design process will thus provide both generic and optional parts and materials.

### Phase IV: Validate

The WIN researchers will use the insights from the phase III workshops, as well as the assessment letters, extensive observations, and participation in the co-design process to describe the final intervention structure. The final intervention will include a program for individual participants, a resource description, a therapist course description, recruitment and course materials, monitoring tools, and an assessment plan. Phase IV involves validating our intervention design with end users and other experts. These include BXs, stakeholders from municipalities, a leading Danish weight-neutral therapist, and fellow researchers. We will present the WIN intervention design and incorporate relevant suggestions.

### Analysis

The data generated in the co-design process will be extensive, including interviews, field notes, e-mails, video-recorded online sessions, informal chats, and photographs. The data will be analyzed in multiple cycles. The initial analysis will be a thematic analysis [50], performed by the core research group, to identify key learning points and how these can be turned into specific intervention elements and activities. In further analysis, we are especially interested in exploring how health is performed, meaning the actions and dialogs that arise when dealing with health guidelines, societal body, and weight and health expectations in the context of the intervention. Here, the work of practice theorist Annemarie Mol becomes relevant, as does her concept of *ontonorms* [41]. By applying this concept, we hope to be able to unfold how the context we live in and the relations we engage in are part of shaping what we view as healthy behavior and what healthy bodies can look like. The WIN intervention will not be promoting weight loss as part of the health intervention for people with BMI>30 kg/m^2^ and instead, we need to know more about how we can promote body liberation, movement, and well-being and motivate participation in the intervention. Here, an unwinding of the expectations, values, and attitudes toward bodies and health will provide valuable information to inform the content of the intervention [51]. There will be a specific focus on the performative element in interviews, workshops, field notes, and observations [50,52].

### Ethical Considerations

This study will be conducted in compliance with the Helsinki Declaration [53]. The study was registered and approved by the ethical committee at the Faculty of Health and Medical Science at the University of Copenhagen (UCPH ethics approval 504-0435/23-5000). Before enrolment, all participants must provide written informed consent explaining all aspects of data production and use. The study will adhere to the General Data Protective Regulation data and information standards. Interviews and observational studies will be captured with a General Data Protective Regulation–compliant app.

Participation in the co-design process is voluntary. Food and drinks will be provided by the project, as well as hotel accommodation when relevant. Participants can claim transport reimbursement. HPs are offered a diploma for their participation, and they are supervised about how to bring input from the study back to their municipality. No economic compensation will be offered to BX or HP, but HPs will be invited to a validation meeting in phase III to help craft specific exercises and provide feedback on intervention elements. A smaller fee will be offered on this occasion.

Because of the use of physical meetings, participants will not be anonymous to one another. Photos can be shared among participants to provide internal feedback. However, all data material (interview transcripts, sound bites, photographs, and video clips) intended for use outside of the project, such as publications and presentations, will be anonymized.

Participation in the project does not increase the likelihood of any severe adverse events. However, some people may be sensitive to working with weight and eating concerns, especially if they have a history of disordered eating. The core research team consists of a trained psychologist specialized in eating disorders and 3 general practitioners who will be alert to any such reactions and provide the necessary support. Adverse events and potential harms will be a focus of discussion at the iteration meetings during the developmental process (see Figure 1). Participants may leave the project at any time. Any unexpected adverse events related to research participation will be reported and included in the study’s final publication.

The project will be widely disseminated through peer-reviewed journals, presentations in relevant professional forums, including municipalities and forums for general practitioners, weight-neutral educational events, academic conferences, and popular dissemination platforms such as podcasts and social media posts.

## Results

The project is fully funded. As of August 2024, the co-design process was in the closing phase. The core research team visited 4 municipalities and learned about the difficulties in implementing a WIN and how these barriers can be worked with. We have included 15 HPs who have dedicated valuable time and energy to our co-design process. A number of them have larger bigger bodies and thus represent a double perspective because they have personal experiences with living with a high BMI as well as their professional experience. Some of the HPs used personal vacation days and others used education possibilities granted by their workplace to be able to participate. In total, 16 BXs have generously shared their experiences with WNH programs, including the difficulties of moving away from external demands and personal wishes for weight loss. We plan to test the intervention for feasibility in 2025.

## Discussion

### Summary

The present protocol has outlined the intervention development process of a co-design of a WNH intervention for use in primary health care settings in Denmark. We aim for the final intervention to be perceived as meaningful to people with BMI>30 kg/m^2^ and that it reduces internalized weight stigma. Relevant stakeholders, including health professionals working with WNH and people with lived experiences, will be invited to participate in the development process.

### Comparison to Existing Literature

A number of earlier pilot studies of WNH interventions have been carried out in the United States and Canada [26,27]. To our knowledge, none of the earlier studies reported a co-design development process, none of the earlier studies have been carried out in a European context, and none have been set in a municipality. Involving HPs and BXs might be effective in identifying key components necessary for a meaningful intervention in a primary health care context. However, the small sample size may limit the generalizability of the findings. The study is conducted within the specific cultural and health care context of Denmark, which may limit the applicability of the findings to other regions with different health care systems or societal attitudes toward weight.

### Limitations

Recruitment for weight reduction studies often faces gender bias, with the majority of participants being women [54,55]. Addressing this limitation is also relevant for this study.

The WNH intervention aims to reduce internalized weight stigma and support participants in connecting with their bodies. A limitation in this respect is that stigma reduction here is bound to the individual participant and—hopefully—the relatives. Weight stigma is a societal problem too big to change in a health intervention, and the change is thus restricted to internalized weight stigma and the development of strategies for the individual to respond to experienced weight stigma, such as negative comments or non-inclusive designs such as the size of seats in public transportation.

### Conclusion and Implications

The expected output of the present co-design project is to gain the needed information to develop a WNH intervention, including elements for specific activities and components to incorporate into the intervention structure. This will form the departure point of a protocol for a weight-neutral feasibility study. The study protocol will be approved by the relevant ethical committees in Denmark. In accordance with the MRC guidelines for complex interventions, the relevant stakeholders who have been part of the development process will be invited to provide feedback on the intervention elements.

The first step will be to conduct a feasibility study focusing on recruitment and retention rates. As part of this feasibility study, a range of psychometric and biological data will be collected on participants as well as qualitative data on motivation and sense-making as part of a WNH program.

Only a few earlier studies of WNH interventions have been designed as randomized controlled trials. They were small and with short follow-ups [28-30]. Our ambition is that the results from the feasibility study will inform a randomized trial with proper dimensions and long follow-up time to shed light on potential health benefits in regard to hard outcomes like cardiovascular events, depression, eating disorders, and mortality. A future RCT will also examine the potential to reduce internalized weight stigma, enhance physical activity, and improve quality of life. We will investigate whether WNH may prove to be a more sustainable way to improve health compared with lifestyle-based weight loss programs.

The intervention setup designed in and from the co-design process, with all activities, components, etc, which are tested in the feasibility study, will in the long term also be available to Danish municipalities free of charge. We will monitor the use of the intervention in the feasibility study, which can inform the RCT study and present novel findings in the field of health management.

## Abbreviations

BX: people with a high BMI
HP: health professional
LightCOM: Lighthouse Consortium on Obesity Management
WIN: weight-neutral intervention
WNH: weight-neutral health
